# Outcome Analysis of Pre-Emptive Embolization of the Collateral Branches of the Abdominal Aorta During Standard Infrarenal Endovascular Aortic Repair

**DOI:** 10.3390/jcm14072391

**Published:** 2025-03-31

**Authors:** Raffaello Bellosta, Francesco D’Amario, Luca Luzzani, Matteo Alberto Pegorer, Alessandro Pucci, Francesco Casali, Mohamad Bashir, Luca Attisani

**Affiliations:** 1 Vascular Surgery–Poliambulanza Hospital, Via L. Bissolati 57, 25124 Brescia, Italyluca.luzzani@poliambulanza.it (L.L.); matteo.pegorer@poliambulanza.it (M.A.P.); alessandro.pucci@poliambulanza.it (A.P.); francesco.casali@poliambulanza.it (F.C.); mohamad.bashir@soutwestwales.ac.uk (M.B.); luca.attisani@poliambulanza.it (L.A.); 2 Vascular and Endovascular Surgery, Velindre University NHS Trust, Health Education & Improvement Wales, Cardiff CF15 7QZ, Wales, UK

**Keywords:** inferior mesenteric artery, embolization, endovascular aortic repair, abdominal aortic aneurysm

## Abstract

**Objectives:** To report the results of pre-emptive embolization of collateral branches of the abdominal aorta in patients undergoing standard bifurcated EVAR versus those undergoing standard EVAR without embolization. **Methods:** This study is a single-center, retrospective, observational cohort analysis of consecutive patients who underwent elective standard endovascular aneurysm repair (EVAR) between 1 October 2013, and 31 December 2022, with a minimum follow-up period of 2 years. The patients were divided into two groups: group A, which did not receive embolization, and group B, which underwent pre-emptive embolization of aortic collateral branches. The primary outcomes for this study include overall survival, freedom from aorta-related mortality (ARM), and freedom from reinterventions related to type 2 endoleak (T2E). In cases of multiple reinterventions, only the first one was considered for this analysis. The secondary outcome focused on assessing freedom from aneurysm sac enlargement. **Results:** We analyzed a total of 265 endovascular aneurysm repairs (EVARs): 183 (69.1%) were classified into group A, and 82 (30.9%) into group B. The median follow-up duration was 48 months [interquartile range (IQR), 28–65.5], which was not significantly different between the two groups [45 months (26–63) in group A vs. 52.5 months (29.5–72.5) in group B, *p* = 0.098]. The estimated cumulative survival rates were 87% (0.2) at 2 years (95% confidence interval [CI]: 82.6–92.9) and 67% (0.3) at 5 years (95% CI: 60.3–73.1), with no significant difference between the groups (*p* = 0.263). The aorta-related mortality rate was 1.1% (*n* = 3); all instances occurred following open conversion due to graft infection (*n* = 2) and in one case of secondary aortic rupture (*n* = 1). In total, 34 cases (12.8%) indicated a secondary intervention related to type 2 endoleak (T2E). The freedom from T2E-related reintervention rate was 99% (0.01) at 2 years (95% CI: 99.4–99.8) and 88% (0.3) at 5 years (95% CI: 81.4–92.5), with no differences between the groups (*p* = 0.282). Cox regression analysis revealed that age over 80 years is an independent negative predictor of survival, with a hazard ratio (HR) of 3.5 (95% confidence interval [CI]: 2.27–5.50; *p* < 0.001). Additionally, T2E-related reintervention was identified as a negative predictor, with an HR of 2.4 (95% CI: 1.05–5.54; *p* = 0.037). In this study, conversion to open repair was necessary for 14 patients (5.3%), with three conversions occurring due to rupture; however, T2E was not a determining factor in any of these conversions. At the last available follow-up computed tomography angiography (CT-A), the median aneurysm diameter was significantly lower in group B, measuring 44 mm (range 37.7–50), compared to group A, measuring 48 mm (range 39–57.5) (*p* < 0.001). Both groups showed a significant change from baseline measurements (*p* = 0.001). **Conclusions:** Pre-emptive embolization of the aortic collateral branches does not lead to improved aorta-related outcomes after EVAR.

## 1. Introduction

Type 2 endoleaks (T2E) can be identified in up to 40% of endovascular aortic repair (EVAR) procedures, either shortly after the intervention or later during follow-up assessments [1]. Despite the prevalence of this complication, the true natural history of T2E remains inadequately understood [2]. In many instances, T2E resolves spontaneously within 6 months; however, reports indicate that persistent or late-onset T2E may be linked to sac enlargement, leading to potential aneurysm rupture or the necessity for further endovascular interventions or surgical conversion [3,4,5]. To mitigate the incidence of T2E and prevent potentially life-threatening complications, the preventive embolization of aortic collateral branches—such as the inferior mesenteric artery (IMA), lumbar arteries, and accessory renal arteries—has been proposed as a strategic approach [6,7,8,9,10,11]. Although recent guidelines from leading vascular societies advocate against routine pre-emptive embolization during EVAR, the evidence supporting its possible benefits is limited and sometimes conflicting [1,12]. This study aims to evaluate the outcomes of pre-emptive embolization of the collateral branches of the abdominal aorta in patients undergoing standard bifurcated EVAR, compared to those undergoing standard EVAR without embolization.

## 2. Materials and Methods

Ethical Statement. The Helsinki Declaration and its later amendments were respected. The study observational study has been submitted to the local Ethics Committee. The data underlying this article are available in the article.

**Figure 1:**
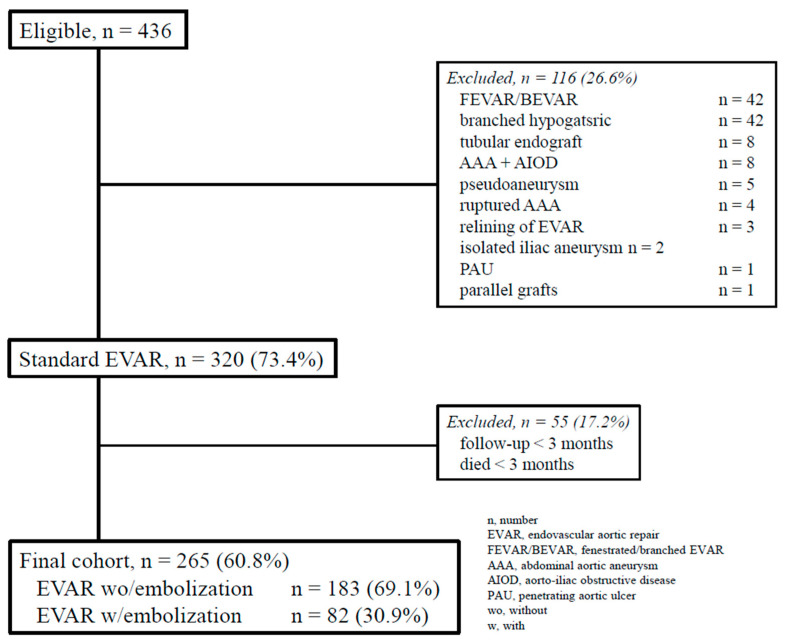
CONSORT algorithm of the patients treated with standard endovascular aortic repair.

Study cohort. This study is a single-center, retrospective, observational cohort analysis. The checklist of items adhered to the STROBE statement [13]. Clinical data were recorded in a dedicated database and analyzed retrospectively. For this study, we included data from consecutive patients treated with endovascular aneurysm repair (EVAR) between 1 October 2013 and 31 December 2022 (Figure 1).

The inclusion criteria were as follows:

- Elective EVAR;
- Follow-up of at least 2 years.

The exclusion criteria were as follows:

- EVAR for symptomatic or ruptured abdominal aortic aneurysm (AAA);
- EVAR for penetrating aortic ulcer, isolated infrarenal dissection, abdominal aortic trauma;
- Complex EVAR (e.g., fenestrated, branched, parallel grafts, endoanchors);
- EVAR performed with tubular endograft (isolate aortic cuff, aorto-uni-iliac);
- Missing clinical or morphologic data;
- Absence of follow-up data.

The information collected included patient demographics, comorbidities, morphologic characteristics of the aortic lesion, anatomic pattern of the aortic collateral branches, and postoperative events (mortality, endoleaks, reinterventions) during hospitalization and follow-up.

### 2.1. Data Availability Statement

The article’s data will be shared on reasonable request to the corresponding author.

### 2.2. Preoperative Workup

All patients underwent preoperative thoraco-abdominal computed tomography–angiography (CT-A) with acquisitions in the arterial and venous phase [14] We used dedicated software (3Mensio^®^—Pie Medical Imaging, version 10.3; NDL) for image reconstruction and volumetric calculation of the AAA sac. Per the institutional approach, data were analyzed by two different operators with >10 years of EVAR experience. Aortic measurements included maximum diameters, patency of the IMA and its diameter estimated at 10 mm from the origin, number of lumbar arteries, and sac thrombus calculated as a percentage, i.e., as the ratio of the area occupied by the thrombus to the area of the aneurysm at the point of maximum transverse diameter.

### 2.3. Operative Indications and Postoperative Surveillance

All interventions were performed according to the national guidelines of the Italian Society for Vascular and Endovascular Surgery (SICVE) and consistent with the most recent clinical practice guidelines on the management of abdominal aorto-iliac artery aneurysms of the European Society for Vascular Surgery (ESVS) [1,13]. For patients included in this cohort, device selection, as well as operative planning, was left to the surgeon’s judgment and was made according to the instructions for use of the manufacturer.

Indications for aortic collateral branch embolization were as follows:

- IMA, when the diameter was >3 mm in diameter;
- Lumbar or sacral arteries, when >2 in number and/or >3 mm in diameter.

Accessory renal artery embolization was performed to obtain an adequate landing zone at the proximal aortic neck or because they originated from the aneurysmal sac. Embolization was performed in all patients the day before EVAR through ultrasound-guided percutaneous radial or common femoral artery access at the operator’s discretion. The collateral branch was engaged through a 4Fr reverse-curved catheter (Bernstein^®^—Cordis; Santa Clara, CA, USA); a 300-cm long floppy 0.014-inch guidewire (Pilot^®^—Abbott; Lake County, IL, USA) was advanced into the collateral branch, followed by a microcatheter (Dirextion™ —Boston Scientific; Marlborough, MA, USA). We consistently used detachable controlled-release coils (Tornado^®^ or Nester^®^—Cook Inc.; Bloomington, IN, USA) or microvascular plugs (MVP™—Medtronic; Minneapolis, MN, USA) for our procedures. For the embolization of the inferior mesenteric artery (IMA), these devices were carefully deployed from the origin of the IMA to just before the left colic artery branch. This approach was taken to preserve collateral circulation to the left colon through the arc of Riolan and the marginal artery of Drummond. We opted not to use liquid agents for the lumbar arteries to prevent peripheral migration and the subsequent risk of spinal cord ischemia.

**Figure 2:**
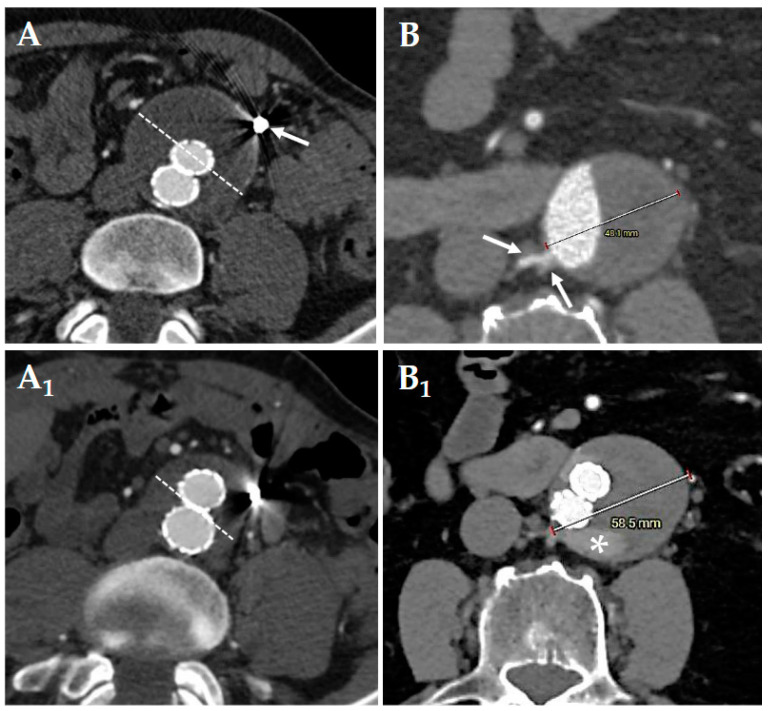
Preoperative (**A**) computed tomography–angiography of pre-emptive embolization of the inferior mesenteric artery ((**A**), white arrow). Postoperative (**A_1_**) follow-up shows a shrinkage of the aneurysmatic sac ((**A**,**A_1_**); sketched white lines). Preoperative (**B**) computed tomography–angiography of standard endovascular aortic repair without lumbar (white arrows) pre-emptive embolization. Postoperative (**B_1_**) follow-up at 2 months shows sac enlargement (48 mm-to-58 mm) due to the presence of a type 2 endoleak with the “nidus” (white asterisk) fed by the pair of lumbar arteries highlighted at the preoperative computed tomography–angiography.

Our follow-up protocol included a CT angiogram (CT-A) within 2 months after endovascular aneurysm repair (EVAR), followed by contrast-enhanced ultrasound (CEUS) every 6 months for the first 2 years and annually thereafter. A new CT angiogram was conducted only if an endoleak was detected or if there was an increase in the aneurysm sac size. During follow-up, we also evaluated transarterial embolization (T2E) in cases of significant sac enlargement (Figure 2).

### 2.4. Definitions and Outcomes

Diameter and volume change were calculated at the last available CT-A, at the time of aortic reintervention, or at the time of death if a definitive imaging study of the endograft (EG) was obtained during the patient’s terminal illness. A persistent T2E was defined if present beyond 6 months after EVAR. Aneurysm sac shrinkage was defined as diameter reduction ≥1 cm according to Society of Vascular Surgery (SVS) reporting standards [14,15]. Significant sac enlargement was defined in the case of ≥5 mm diameter enlargement in comparison with the baseline preoperative CT-A. The cause of death was classified as verified only when based on autopsy findings, direct surgical observation, or imaging studies obtained during the patient’s terminal illness. The follow-up index (FUI) describes the completeness of follow-up at a given study end date and the ratio of the period investigated to the potential follow-up period [16]. The study closed on 31 December 2024: information on the aorta-related reintervention, vital status, and date of death of the individual patient was validated by death certificates, electronic records maintained by the regional health system, interview with the general practitioner, or data certified by admission to the emergency room.

The primary outcomes for this specific study were overall survival, freedom from aorta-related mortality (ARM), and freedom from T2E-related reintervention.

In the case of multiple reinterventions, this latter was calculated at the first one. The secondary outcome was the assessment of freedom from aneurysm sac increase.

### 2.5. Statistical Analysis

Clinical data were collected prospectively in a single database, recorded, tabulated in Microsoft Excel (Microsoft Corp, Redmond, Wash), and analyzed retrospectively [17]. Statistical analysis was performed with spss, release 29.0 for windows (IBM SPSS Inc.; Chicago, IL, USA). Continuous variables were tested for normality using Shapiro–Wilk’s test and compared between groups with unpaired Student’s *t*-test for normally distributed values; otherwise, the Mann–Whitney U test was used. Variables normally distributed are presented as mean ± standard deviation (SD) and range; otherwise, they are presented as median and 25th–75th interquartile (IQR). Categorical variables are presented using frequencies and percentages and were analyzed with Pearson’s χ^2^ test or Fisher’s exact test to determine whether the expected cell frequencies were <5. The Wilcoxon signed-rank test was used to evaluate the difference in covariate measurements before and after EVAR. Multivariable analysis was used to adjust the relationship between the type of EVAR and 30-day mortality and survival, as well as T2E-related reintervention or sac increase. Associations that yielded a *p* value < 0.20 in a univariate screen were then included in a binary logistic regression analysis using Wald’s forward stepwise model. The strength of the association of variables with each primary outcome was estimated by calculating the odds ratio (OR) and 95% CI (95%CI): significance criteria 0.20 for entry, 0.05 for removal. Follow-up freedom from ARM and EVAR-related reintervention rates were estimated according to the Kaplan–Meier method and reported with standard error (SE) and associated 95%CI. The Breslow rank test was used to make any possible comparisons in the follow-up of the different covariates. Time-dependent coefficients were included in Cox proportional hazards regression and survival analysis

In addition, the need for T2E-related reintervention was estimated with a proportional hazards model proposed by Fine and Gray to consider the presence of competitive risks. All reported *p* values were two-sided; a *p* value < 0.05 was considered significant.

## 3. Results

### 3.1. Study Cohort

Out of a total of 436 EVAR procedures, 265 (60.8%) were included in the final analysis. Among these, 183 patients (69.1%) underwent EVAR without prior embolization (designated as group A), while 82 patients (30.9%) received pre-emptive embolization during the EVAR procedure (designated as group B). Table 1 presents demographic data and comorbidities. Notably, group A had a higher proportion of female patients [*n* = 20 (10.9%) compared to 2 (2.5%); odds ratio (OR): 3.6, *p* = 0.028], as well as a higher median age [77 years (interquartile range, IQR: 70–81) vs. 73.5 years (67–77), *p* = 0.001].

The majority of patients [*n* = 238 (89.8%)] had a patent inferior mesenteric artery (IMA), while 14 patients (5.3%) had a mixed pattern of vessels (including IMA, lumbar, and/or accessory renal), and 13 patients (4.9%) had only patent lumbar arteries. Regarding anatomic measurements, the median IMA diameter was significantly larger in group B [2.7 mm (2.3–3) in group A vs. 3.6 mm (3.3–4) in group B; *p* < 0.001]. No other significant differences were found between the two groups (Table 2).

The number of patients with more than three pairs of lumbar arteries was similar in both groups [*n* = 164 (89.6%) in group A vs. 76 (92.7%) in group B; OR: 1.5, *p* = 0.502]. In group B, 64 cases (78.0%) involved one vessel embolization, while 18 cases (22.0%) involved the embolization of two or more vessels.

### 3.2. Outcomes Analysis

Operative mortality was never observed. Technical success was achieved in all cases. Visceral or spinal cord ischemic complications correlated with pre-emptive embolization did not occur. The median follow-up was 48 months (IQR, 28–65.5), and it was not different between the two groups [45 (26–63) vs. 52.5 (29.5–72.5), *p* = 0.098]. The mean follow-up index was 0.7 ± 0.3 (range, 0–1), and it was not different between the two groups (0.65 ± 0.3 vs. 0.70 ± 0.2, *p* = 0.158).

a. survival

**Figure 3:**
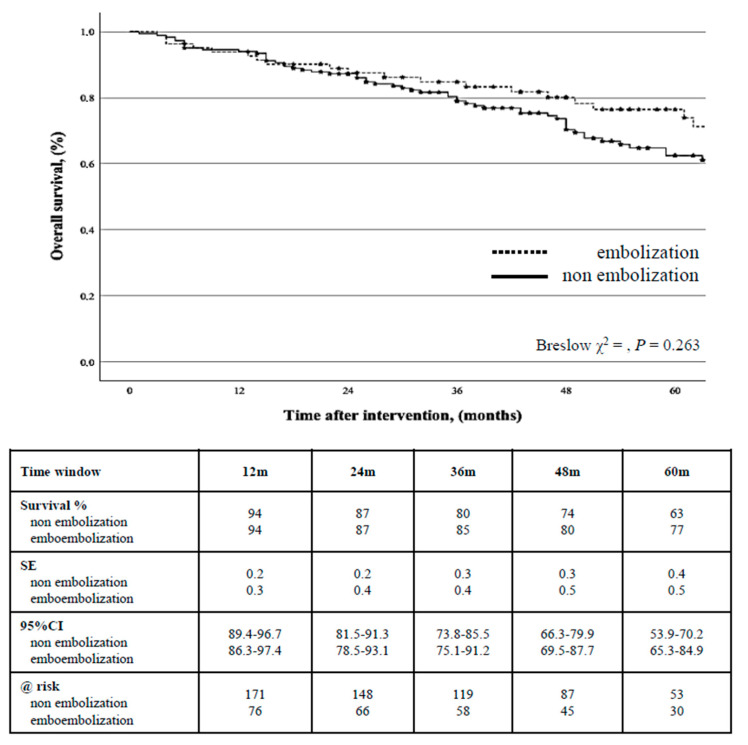
Kaplan-Meier estimate of cumulative survival stratified by type of endovascular aortic repair strategy.

During the follow-up, 81 (30.6%) patients died; the estimated cumulative survival was 87% (0.2) at 2 years (95%CI: 82.6–92.9) and 67% (0.3) at 5 years (95%CI: 60.3–73.1), with no difference between the groups (*p* = 0.263) (Figure 3).

The aorta-related mortality rate was 1.1% (*n* = 3): all of them occurred after open conversion due to EG infection (*n* = 2) or secondary aortic rupture (*n* = 1). The univariate screen identified that age > 80 years and aneurysm sac increase during the follow-up were associated with survival; however, Cox’s regression analysis identified only age > 80 years as an independent negative predictor of survival (HR: 3.5, 95%CI: 2.27–5.50, *p* < 0.001, Table 3), and it was associated with this outcome even when stratified by type of EVAR strategy (*p* < 0.001).

b. reintervention for endoleak

**Figure 4:**
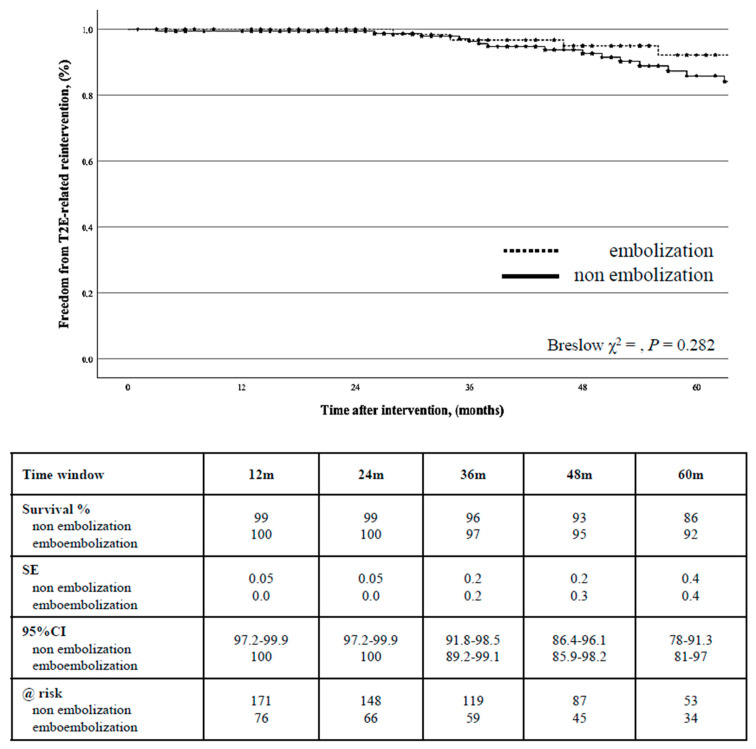
Kaplan-Meier estimate of freedom from type 2 endoleak-related reintervention stratified by type of endovascular aortic repair strategy.

A T2E-related reintervention for endoleak was indicated in 34 (12.8%) cases. The freedom from T2E-related reintervention rate was 99% (0.01) at 2 years (95%CI: 99.4–99.8) and 88% (0.3) at 5 years (95%CI: 81.4–92.5): there was no difference between the groups (*p* = 0.282) (Figure 4).

Univariate screening identified that age > 80 years, smoking habit, conic shape of the proximal aortic neck, and the presence of more than three pairs of patent lumbar arteries were associated with T2E-related reintervention: in Cox’s regression analysis, only age > 80 years (HR: 2.4, 95%CI: 1.05–5.54, *p* = 0.037, Table 3) was associated with this outcome, even when stratified by type of EVAR strategy (*p* = 0.048).

c. conversion to open repair

Conversion to open repair was necessary in 14 (5.3%) patients. Secondary aortic rupture occurred in three (1.1%) cases: it was never determined by T2E but always correlated with type 1 endoleak. In three (1.1%) cases, the indication for open conversion was EG infection, but only one occurred with type 1 endoleak. The freedom from open conversion rate was 99% (0.05) at 2 years (95%CI: 97.5–99.7) and 95% (0.3) at 5 years (95%CI: 90.3–97.6): there was no difference between the groups (*p* = 0.858). No covariate was associated with conversion to open repair in Cox’s regression analysis.

d. sac evolution

There were no differences between the groups either in terms of sac shrinkage (*p* = 0.783) or sac enlargement (*p* = 0.239). We detected 98 (37.0%) endoleaks, 23 (8.7%) type 1 and 72 (27.9%) type 2, while in one (0.4%) case, we observed a combination of the two types. No type 3 endoleak was observed. The types of endoleak were not different between the groups (*p* = 0.847). At the last available CT-A, the median aneurysm diameter was smaller in group B [mm, 48 (39–57.5) vs. 44 (37.7–50), *p* < 0.001] with a significant change from the baseline measurement in both groups (*p* = 0.001). Stability of the sac or any sac diameter decrease was observed in 166 (62.6%) cases; an increase in sac diameter was detected in 49 (18.5%) cases, and there was a significant enlargement in 35 (13.2%). Univariate screening identified that age > 80 years, smoking habit, hypertension, and the presence of more than three pairs of patent lumbar arteries were associated with sac enlargement, but Cox’s regression analysis did not identify significant association with any of these variables; only age > 80 years (HR: 2.1, 95%CI: 0.97–4.62, *p* = 0.058, Table 3) was shown to increase the risk of sac enlargement.

## 4. Discussion

The main findings of our analysis are as follows: pre-emptive embolization of the aortic collateral branches during endovascular aneurysm repair (EVAR) does not seem to protect against complications related to EVAR or the need for reintervention. Additionally, being over 80 is the most significant predictor of major aorta-related outcomes.

Since the early days of the EVAR technique, numerous studies have identified aorta-related reinterventions as a major drawback, particularly during long-term follow-up. These reinterventions are primarily associated with the development and consequences of persistent type 2 endoleaks (T2E) [18,19]. Research indicates that pre-emptive embolization may lower the risk of type 2 endoleaks (T2E) in the mid-term. However, this potential benefit does not seem to reduce the likelihood of reinterventions related to endovascular aneurysm repair (EVAR) [6,7,8,9,10,11]. Our experience aligns with these findings. Despite observing a high rate of positive remodeling in the aneurysmal sac, characterized by significant shrinkage, pre-emptive embolization did not prevent T2E-related reinterventions. There is plenty of literature that reported several different predictors of reintervention, with age > 80 years being the most important in our series [20]. Giving an unquestionable explanation of why ageing patients should be more prone to T2E-related reintervention is impossible at this time; nevertheless, our data may find robust support in several experiences that reported a significantly increased risk of reintervention after EVAR in octogenarians [21,22,23,24,25,26]. Furthermore, age is an important issue in all surgical scenarios because older patients generally have a higher operative risk. As far as octogenarians are concerned, our experience confirms the results in the literature showing that life expectancy in octogenarians was significantly poorer [23]. Considering the higher reintervention rate and the fact that a greater proportion of the population lives longer, factors such as life expectancy and risks involved in the procedure may increase the attention toward careful patient selection, especially in the older cohort.

In evaluating the risk of reintervention, anatomic variables may play an important role, especially after EVAR. Among the several covariates that have been associated with the risk of reintervention, the diameter of the IMA, the number of patent lumbar arteries, and the proportion of maximum aneurysm area occupied by thrombus have been identified as being associated with a higher risk of a persistent T2E [27,28,29,30,31]. In those anatomic circumstances, pre-emptive embolization has been suggested to be potentially useful to limit the occurrence of persistent T2E and was initially intended to protect against life-threatening complications such as rupture and/or the need for open conversion [6,18] Our analysis shows that pre-emptive embolization of aortic collateral branches does not confer better protection against reintervention and open conversion, notwithstanding the positive remodeling of the aneurysmatic sac. However, the addition of another embolization strategy, such as sac filling, failed to prevent persistent T2E occurrence and reinterventions. Nonetheless, we can re-evaluate the glass-half-full nature of this circumstance; secondary ruptures and aorta-related mortality never occurred in the case of persistent T2E, so this condition should not be considered a malignant condition, as some authors have considered it [14,32,33,34]. Therefore, all these data support the recent ESVS guidelines that advised against any kind of routine additional pre-emptive embolization during EVAR [1,12].

## 5. Conclusions

Our analysis appears to substantiate that pre-emptive embolization of the aortic collateral branches during endovascular aortic repair (EVAR) does not yield improved aorta-related outcomes. Additionally, advanced age, specifically exceeding 80 years, emerges as a significant predictor of inferior outcomes. Furthermore, although embolization did not prove effective in reducing the need for reintervention, the presence of persistent type 2 endoleak (T2E) should not be classified as a malignant condition, given the lack of instances of secondary rupture or a markedly elevated risk of related reinterventions.

## Figures and Tables

**Table 1:** Demographic data, comorbidities and risk factors of the patients stratified by type of EVAR strategies.

	Entire Cohort	Group A	Group B	OR	*p*
	(*n* = 265)	(*n* = 183)	(*n* = 82)		
Demographics					
M:F ratio	243:22	163:20	80:2	3.6	0.028
Age, median (IQR)	76 (70–79)	77 (70–81)	73.5 (67–77)		0.001
>80 years	65 (24.5)	55 (30.0)	10 (12.2)	2.3	0.002
Comorbidities, *n* (%)					
Hypertension	207 (78.1)	141 (77.0)	66 (80.5)	1.2	0.630
Coronary artery disease	91 (34.3)	64 (35.0)	27 (32.9)	0.9	0.781
Diabetes	35 (13.2)	22 (12.0)	13 (15.8)	1.4	0.434
Smoking habit	78 (29.4)	51 (27.9)	27 (32.9)	1.3	0.466
Antithrombostic regimen					
Antiplatelet therapy	183 (69.1)	128 (69.9)	55 (67.1)	0.9	0.668
Oral anticoagulant	32 (12.1)	26 (14.2)	6 (7.3)	0.5	0.163

*n* = number; OR = odds ratio; IQR = interquartile range; M = male; F = female.

**Table 2:** Anatomic features of the abdominal aortic aneurysms stratified by type of EVAR strategy.

	Entire Cohort	Group A	Group B	OR	*p*
	(*n* = 265)	(*n* = 183)	(*n* = 82)		
Aortic features, median (IQR)					
Maximum diameter (mm)	54 (51–60)	54 (50–60)	54 (52–58)		0.210
Neck length (mm)	22 (17–30)	25 (20–30)	25 (18–30)		0.773
Neck angulation (degrees)	15 (5–27.5)	15 (5–31.7)	15 (5–25)		0.367
Conic shape, *n* (%)	67 (25.3)	49 (26.8)	20 (24.4)	0.8	0.648
Thrombus rate (%)	40 (22.7–60)	38.5 (20.5–60)	35 (18–50)		0.135

EVAR = endovascular aortic repair; *n* = number; OR = odds ratio; IQR = interquartile range.

**Table 3:** Univariate screen and multivariate analyses of cumulative survival and freedom from T2E-related reintervention.

	Overall Survival				
	Univariate		Multivariable		
Covariate	Log-rank		HR	95%CI	*p*
Age > 80 years	<0.001		3.5	2.27–5.50	<0.001
Sac increase	0.180				
	Freedom from T2E-related reintervention				
	Univariate		Multivariable		
Covariate	Log-rank		HR	95%CI	*p*
Age > 80 years	0.007		2.4	1.05–5.54	0.037
Smoking habit	0.024				
Conic shape	0.046				
>3 lumbar	0.078				

T2E = type 2 endoleak; HR = hazard ratio; 95%CI = 95% confidence interval.

## Data Availability

The data underlying this article will be shared on reasonable request to the corresponding author.
